# Optimal Dosing Regimen of Osteoporosis Drugs in Relation to Food Intake as the Key for the Enhancement of the Treatment Effectiveness—A Concise Literature Review

**DOI:** 10.3390/foods10040720

**Published:** 2021-03-29

**Authors:** Agnieszka Wiesner, Mariusz Szuta, Agnieszka Galanty, Paweł Paśko

**Affiliations:** 1 Department of Food Chemistry and Nutrition, Faculty of Pharmacy, Jagiellonian University Medical College, 9 Medyczna Str., 30-688 Kraków, Poland; agnieszka.wiesner@doctoral.uj.edu.pl; 2 Department of Oral Surgery, Faculty of Medicine, Jagiellonian University Medical College, 4 Montelupich Str., 31-155 Kraków, Poland; mariusz.szuta@uj.edu.pl; 3 Department of Pharmacognosy, Faculty of Pharmacy, Jagiellonian University Medical College, 9 Medyczna Str., 30-688 Kraków, Poland; agnieszka.galanty@uj.edu.pl

**Keywords:** bisphosphonates, SERMs, interaction, food, supplements, bioavailability, meal, coffee, juice, mineral water

## Abstract

Bisphosphonates and selective estrogen receptor modulators (SERMs) represent the two most important groups of medications taken orally and employed in osteoporosis treatment. Effectiveness of the therapy may be affected by poor patient adherence, in particular, due to the inconvenient dosing regimen of oral bisphosphonates. With this review we aimed to assess the effects that food, beverages, and dietary supplements consumed during treatment, along with the dosing regimens, may have on pharmacokinetics and pharmacodynamics of oral drugs employed in treating osteoporosis; we also aimed to shape the recommendations valuable for professional patients’ counseling and education, to provide appropriate dosing regimens in order to improve adherence to the therapy. Food, beverages such as coffee, juices, and mineral water, as well as dietary supplements containing multivalent cations, e.g., calcium, magnesium, aluminium, iron, showed to have a deleterious effect on the bioavailability of all the investigated oral bisphosphonates, specifically alendronate, risedronate, ibandronate, minodronate, and etidronate. For risedronate, a delayed-release (DR) tablet was designed to solve the malabsorption problem in the presence of food, hence DR risedronate can be ingested following breakfast. For other oral bisphosphonates, the proper interval between drug and food, beverages, and dietary supplements intake should be maintained to minimize the risk of interactions. The effect of food on pharmacokinetic parameters of selective estrogen receptor modulators (SERMs) was found to be clinically irrelevant.

## 1. Introduction

Being a chronic skeletal disease that leads to a gradual bone density loss and increased bone fragility, as well as susceptibility to fractures, osteoporosis remains a significant reduced life quality risk factor in older patients. At least one in three women and one in five men in their 50s are estimated to experience osteoporotic fracture in their lifetime, often followed by further serious health consequences. Due to the progressive population aging, the problem of treating osteoporosis is more actual than ever. To provide the effective therapy with the two most important groups of drugs used in osteoporosis treatment, namely bisphosphonates and selective estrogen receptor modulators (SERMs), their proper administration in relation to food/meal time should be followed. Poor patient’s adherence to drug administration regimens is considered one of the main problems that may affect the efficacy of the therapy, as it depends on the correct medications administration schemes and persistence in applying the treatment [1]. It is estimated that 50% of all patients discontinue therapy with oral bisphosphonates within 1 to 2 years from onset [2], or even earlier, before any clinical effect can be seen [3]. Moreover, even one-third of the patients may administer their medications improperly [4]. It is impossible to indicate one reason for poor adherence, as the problem is multifaceted. Yeam et al. [5] recognized up to 24 factors that can affect medications adherence among patients with osteoporosis. However, in many studies, top two issues reported by patients as the cause of non-adherence to osteoporosis treatment seem to predominate. The first issue is the poor motivation to take the drugs, due to the lack of disease symptoms and hence no clinical signs of drug effectiveness. The second problem includes inconvenient dosing regimen of oral bisphosphonates, especially the need to fast before, and to stay upright after the administration [2,3,4]. In response to the problem of non-adherence, Cornelissen et al. [6] concluded recently that patient education, supervision, counselling, and shared decision making on changing the dosing regimen, may improve both persistence and compliance, and consequently, also the effectiveness of osteoporosis treatment.

Another general problem is that patients, physicians, pharmacists and dieticians may underestimate the impact of food and dosing regimen on drugs effectiveness [7]. A recent study in a district general hospital suggests that healthcare professionals shall be reminded about proper dosing regimens for oral bisphosphonates. In an observation period of 6 months, Wilcock et al. [8] indicated that among 398 doses of alendronate or risedronate administered to patients in a hospital setting, half of the doses were given less than 30 min before breakfast, that is at the time when food may dramatically reduce bisphosphonates absorption. Although the main limitation of the study was the assumption around the exact time of breakfast, these results should be taken into consideration. 

In elderly patients, the greater emphasis should be put on food-drug interactions due to the polypharmacy, and self-medication with dietary and nutritional supplements and OTC drugs (including herbal medicines) [9]. Thus, this review focused on the effects that consumption of food, beverages, and dietary supplements, as well as dosing regimens, may have on pharmacokinetics and pharmacodynamics of oral drugs employed in treating osteoporosis. This is the first such comprehensive review that brings together the information not only about bisphosphonates, but also about selective estrogen modulators, with the emphasis on oral forms of the drugs. Some valuable recommendations, for professional patients’ counselling, summarizing the intake of the osteoporosis drugs with relation to food, and presenting how to avoid the potential interactions, are also included in the review.

## 2. Materials and Methods

To collect information on interactions between food and medications for osteoporosis, the authors, namely AW and PP, performed a literature research in the Medline (via PubMed) and Embase databases, covering reports from 1986 to 2021. The following keywords and phrases were applied in the searching process: drugs names (“alendronate”, “risedronate”, ”ibandronate”, “minodronate”, “etidronate”, “raloxifene”, “bazedoxifene”) in combinations with “food”, “food-drug interaction”, “meal”, “breakfast”, “bioavailability”, “juice”, “coffee”, “tea”, “milk”, “iron”, “aluminum”, “calcium”, “magnesium”, “alcohol”, and “mineral water”. Furthermore, other resources such as drugs.com, Micromedex, AHFS, and UpToDate, were also researched, as well as characterization charts of particular medicinal products. Additional publications were found by checking the reference lists. 

**Figure 1:**
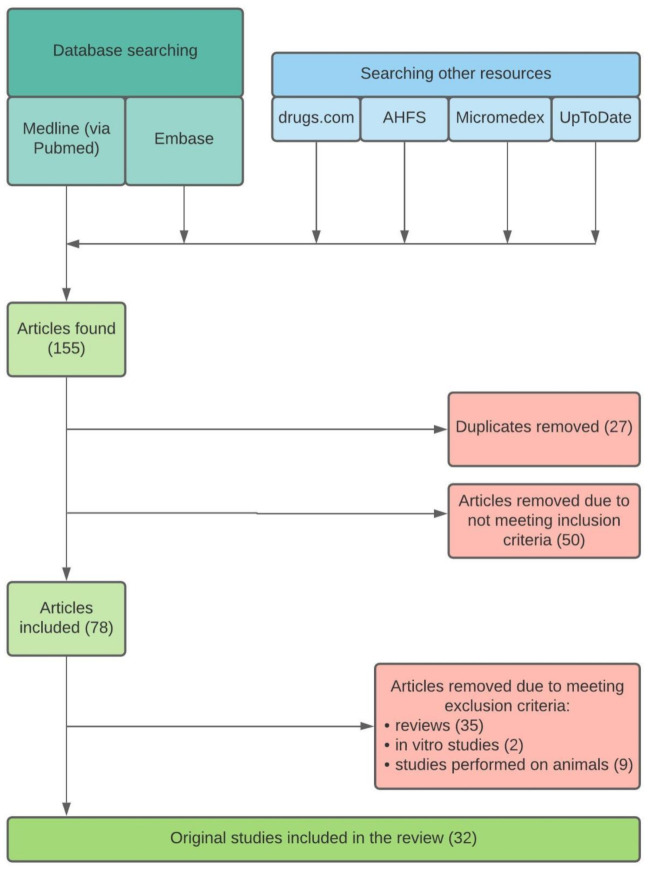
Searching strategy flowchart.

Initially, 155 articles were tracked. After removing 27 duplicates and performing the first evaluation, 78 articles reporting or investigating the effect of meals, beverages, dietary supplements, and dosing regimen on pharmacokinetics and pharmacodynamics of drugs used in oral osteoporosis treatment were considered to be included. No restrictions for study design, participants’ characteristics, or sample size were made. 35 reviews, two in vitro studies, and nine studies performed on animals were subsequently excluded. Finally, 32 original studies remained included and are discussed in this review. A flowchart of the searching strategy is presented in Figure 1.

## 3. Results and Discussion

### 3.1. Oral Bisphosphonates

Various guidelines on managing osteoporosis recommend oral bisphosphonates as the first-choice drugs [10,11,12,13], widely prescribed in postmenopausal osteoporosis, secondary osteoporosis, and male osteoporosis. Bisphosphonates are often referred to as “antiresorptive drugs”, due to their mechanism of action, as they inhibit osteoclast-mediated bone resorption [14]. A recent meta-analysis confirmed bisphosphonates’ use to decrease the risk of both vertebral and non-vertebral osteoporotic fractures [15]. The treatment becomes effective relatively early, namely within 6–12 months from starting the therapy, and the results tend to prolong [16]. Despite its established effectiveness, therapy with oral bisphosphonates may still be found challenging, due to the overall low bisphosphonates bioavailability, likely gastrointestinal side effects, such as heartburn, irritated esophagus, nausea, gastric ulcers, etc., but also the potential interactions with food and dietary supplements [14,16]. 

#### 3.1.1. Chemical Characterization of the Bisphosphonates

**Figure 2:**
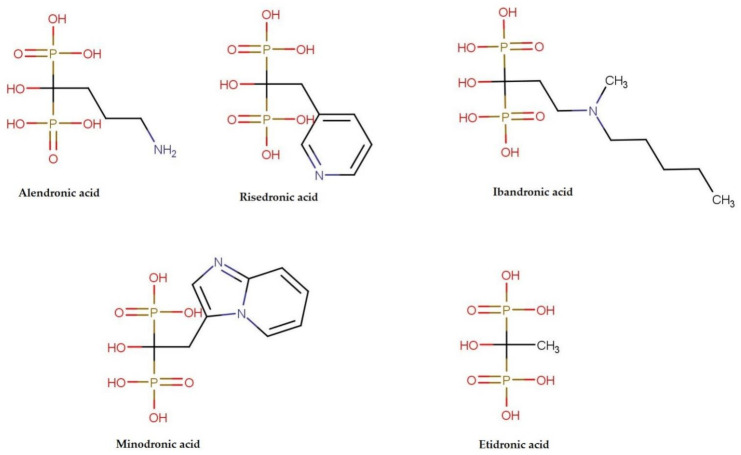
Chemical structures of bisphosphonates discussed in the review.

All bisphosphonates are the analogs of pyrophosphate, containing two phosphate groups linked by carbon atom (P-C-P), instead of an oxygen atom [17]. Additionally, as presented in Figure 2, each bisphosphonate has two side chains bound to the central carbon atom.

Of the drugs discussed in this review, only etidronate does not contain nitrogen atom, as it is representative of the oldest, first-generation of bisphosphonates. The addition of nitrogen, either as a constituent of the amine group (alendronate and ibandronate) or heterocyclic ring (in risedronate and minodronate) is known to enhance the antiresorptive activity [17]. 

The Biopharmaceutical Classification System (BCS) assigns each pharmacologically active compound to one of four classes, based on its water solubility and intestinal membrane permeability, since these parameters determine the rate and extent of drug absorption [18]. Bisphosphonates, as the highly soluble and hardly permeable compounds, belong to the III class of BCS. High water solubility (in other words—very low lipophilicity) of bisphosphonates is due to the presence of hydrophilic phosphate groups. These groups are negatively charged in physiological pH, hence the bisphosphonate compound is completely ionized in the intestine lumen. Ionized and hydrophilic compounds penetrate lipid membranes with great difficulty. That explains both the low permeability and very low oral bioavailability of bisphosphonates, which is known to range from 0.6 to 3%, depending on the drug [19].

#### 3.1.2. Interaction with Di- and Trivalent Cations

Although the overall effect of calcium, or vitamin D alone, on the reduction of fracture risk remains unclear [13,20], the supplementation of both substances is currently recommended for patients at high risk of calcium and vitamin D insufficiency, and in those treated for osteoporosis [21]. In consequence, concomitant use of oral bisphosphonates and calcium preparations is widespread. 

Oral bisphosphonates are chelating agents that form salts with multivalent cations present in food, dietary supplements, drugs (e.g., antacids), and mineral water [14]. These salts are insoluble at pH > 5, and as such, bisphosphonate cannot be absorbed in the intestine [22]. In characterization charts of oral bisphosphonates, the recommended minimum time interval between their ingesting and the intake of vitamins, dietary supplements or antacids, containing multivalent cations such as calcium, aluminum, magnesium, and chromium, varies from at least 30 min to 2 h, depending on the drug [23,24,25,26,27], while at least last 2 h for iron preparations [28]. For oral bisphosphonates taken once a week, or once a month, it may be even justified to refrain from administering calcium preparations on the same day as a bisphosphonate. 

To improve the patients’ understanding of co-treatment rules, but also compliance, fixed-combination packs of bisphosphonates and calcium carbonate were designed and tested. In a cross-over study in 164 postmenopausal women, Ringe et al. [29] revealed that use of the combination pack containing one tablet of risedronate 35 mg and six tablets of calcium carbonate 1250 mg (500 mg of elemental calcium) for one week, significantly improved patients’ comprehension of dosing instructions, when compared to separate packaging (80% vs. 70%, respectively). In some countries such fixed-combinations packs are already registered, e.g., for alendronate (*APO-Alendronate Plus D3 and calcium*), or risedronate (*Actonel Combi*).

#### 3.1.3. Problem with Water Type

The results of preclinical studies indicated that alendronate and risedronate absorption may decrease with the increasing concentration of calcium in mineral water used for drug administering [30,31]. As instructed by prescribing guidelines, oral bisphosphonates are recommended to be taken with tap water, to minimize the risk of malabsorption caused by calcium and other cations present in mineral and spring waters. However, tap water can be alkaline as well. Pellegrini et al. [32] reported that in some regions of Italy (e.g., Rome or Milan) calcium content of tap water might be even up to 100 times higher than in some commercially available bottled waters (100–110 mg/L vs. 1–2 mg/L, respectively). Morr et al. [33] obtained similar results after examining tap waters from cities in the USA and Canada, where reportedly, calcium content varied from 1 to 135 mg/L. For comparison, spring waters were found to contain lower calcium concentrations (approximately 21.8 mg/L), and mineral waters in general showed higher calcium content (an average of 208 mg/L). Due to the lack of experimental evidence, neither specific nor approximate maximum calcium intake that would not alter bisphosphonates absorption has been established. For that reason, Azoulay et al. [34] emphasized the need to check the mineral content of both bottled and tap waters before administering bisphosphonates. Moreover, Morr et al. [33] observed that filtering tap water with a home filter system may remove on average 89% of calcium, hence proposed this alternative for patients living in regions with the high calcium content of tap water. Such approach may be thus recommended as one of the elements of preventing the decrease in the effectiveness of bisphosphonates therapy.

#### 3.1.4. Alendronate

Alendronate (syn. alendronic acid, alendronate sodium) is available in tablet (*Fosamax*, *Ostemax*), effervescent tablet (*Binosto*), and oral solution (*Fosamax*) forms, taken daily or weekly. The same doses of different alendronate formulations are bioequivalent [23,35]. Additionally, in 2012, the oral jelly formulation (*Bonalon*) has been approved in Japan, to reduce the risk of choking and to prevent gastrointestinal side effects [36]. In some countries combination of alendronate in the same tablet with vitamin D (*Fosamax Plus*) is registered as well. 

Oral bioavailability of alendronate under fasting conditions is 0.64% in women (for dosage range 5–70 mg), and 0.59% in men (for dose 10 mg); in the presence of food and beverages further decrease is observed [23,37]. 

Gertz et al. [22] conducted series of open-label, randomized, cross-over trials to establish the most optimal regimen for alendronate administration regarding food and beverages. In the first study of 10 post-menopausal women, a single dose of 20 mg alendronate tablet was administered after an overnight fast, in three dosing regimens: (1) 2 h before breakfast—reference regimen, (2) 1 h before breakfast, or (3) 30 min before breakfast; with or without calcium carbonate supplement (containing 1000 mg of elemental calcium). The detailed composition of the breakfast was described in Table 1—Set No 1. In the second study of 49 post-menopausal women, a single dose of 10 mg alendronate tablet was taken after an overnight fast, in five dosing regimens: (1) 2 h before breakfast—reference regimen, (2) 1 h before breakfast, (3) 30 min before breakfast, (4) immediately after breakfast, or (5) 2 h after breakfast (Table 1, Set No 2). Results of both studies were comparable and found that the administration of alendronate 30 min and 1 h before breakfast reduced its bioavailability approximately by 40%, relative to the reference regimen. Moreover, the second study revealed that ingesting alendronate concomitantly with breakfast, or even 2 h after the meal, may drastically impair drug absorption (by 85–90%). Adding calcium supplement to breakfast in the first study did not cause an additional decrease in absorption when compared to ingesting the meal alone. In the third study of 40 post-menopausal women, a single dose of 10 mg alendronate tablet was taken, followed an overnight fast, 2 h before breakfast, with (1) 240 mL of tap water—reference regimen, (2) black unsweetened coffee, or (3) orange juice. Coffee and orange juice were found to significantly reduce alendronate absorption (by 60% for both beverages) relative to administration with water. What is worth noting, the study is probably the only example, describing the interactions between any bisphosphonate and coffee and fruit juice, thus the important results should be further continued to draw more reliable conclusions. 

Wagener et al. [38] assessed whether the ingestion of alendronate during the day, with 1 h fast before a meal, could be an effective dosing regimen. After 8 weeks of study, significant changes in biochemical markers of bone turnover, namely osteocalcin, alkaline phosphatase, and dipeptidyl peptidase, were observed, suggesting that despite a non-optimal dosing regimen, the efficacy of treatment was maintained.

The impact of food on the bioavailability of alendronate was investigated for effervescent tablet formulation as well. A study of 119 healthy women revealed that taking 70 mg alendronate 15 min before standard breakfast may decrease its bioavailability by approximately 50%, when compared to administering 4 h before a meal [35]. 

Interestingly, a recent prospective case-control study [2] suggested that administering alendronate after breakfast, despite the existing evidence of the impaired drug absorption for this regimen, may have positive influence on a patient’s persistence and adherence, and hence improve the efficacy of treatment. Park et al. [2] compared 1-year persistence and therapy effectiveness in patients taking 5 mg alendronate tablet in two regimens: (1) according to the guidelines. i.e., 30 min before breakfast, and (2) after breakfast. After 1 year, the medication possession ratio (MPR), measured as the sum of administration days divided by 365 days, was significantly higher in patients taking alendronate after breakfast, relative to the recommended regimen (0.71 vs. 0.66, respectively), which indicate enhanced persistence and adherence. Additionally, significant improvement in lumbar and hip T-scores was observed in both groups, together with no significant differences between the groups, which suggests the therapy effectiveness to be similar for both treatment regimens.

#### 3.1.5. Risedronate

Risedronate (syn. risedronic acid, risedronate sodium) is available in two formulations: immediate-release (IR) tablets (*Actonel*, *Risendros*) taken daily, once a week or once a month, and delayed-release (DR) tablets (*Atelvia*) taken once a week. 

##### Immediate-Release (IR) Tablets

The mean oral bioavailability of the 30 mg IR tablet of risedronate is 0.63%, when administered while fasting, whereas the concomitant intake of food decreases it further [24]. Mitchell et al. [39] conducted a randomized, parallel study in 127 healthy volunteers, to compare four different timing regimens for risedronate administration: (1) after night fasting, 4 h before lunch (Table 1, Set No 3) that was the reference regimen, (2) after night fasting, 1 h before a high-fat breakfast (Table 1, Set No 4), (3) after night fasting, 30 min before a high-fat breakfast (Table 1, Set No 4), (4) 2 h after a standard dinner (Table 1, Set No 5). After administering a single dose of 30 mg risedronate IR tablets in each group, pharmacokinetic parameters such as AUC_0–∞_ and C_max_ were measured. Risedronate, both taken 30 min before breakfast (group 3), and 2 h after dinner (group 4), showed the lowest extend of absorption, when compared to the reference regimen; AUC and C_max_ were reduced by 56 and 32% in group 3; and by 52 and 75% in group 4, respectively. To compare, when risedronate was taken 1 h before breakfast (group 2), AUC and C_max_ were reduced by 32 and 14%, respectively. Mitchell et al. concluded that although the most optimal dosing regimen for risedronate in IR tablets is 1 h before breakfast, a flexible-dosing approach may be considered, since dosing either 30 min before breakfast or 2 h after dinner resulted in a similar extend of risedronate absorption.

Ogura et al. [12] performed a similar study in 12 healthy Japanese volunteers, randomly assigned to administer 5 mg of IR risedronate (1) under fasting conditions, without breakfast—a reference regimen, (2) 30 min before breakfast, (3) 30 min after breakfast, and (4) 3 h after breakfast. Breakfast contained 200 mg of elemental calcium. To compare the level of risedronate absorption in different regimens, such pharmacokinetic parameters as AUC_0–24_, C_max_, t_max_, and t_1/2_ were measured. Administering risedronate 30 min after breakfast (3) caused the highest decrease in AUC_0–24_ and C_max_ (both by 94%), and the highest increase in t_max_ and t_1/2_ (by 62 and 39%, respectively), when compared to the reference regimen. Contrastingly, administration 30 min before breakfast decreased AUC_0–24_ by 63%, and C_max_ by 26%, hence Ogura et al. recommended this regimen as the most optimal for Japanese patients. However, it should be emphasized that in this study administration 60 min before breakfast was not investigated. 

In two studies of a total number of 1582 postmenopausal women with osteoporosis, Kendler et al. [41] assessed the influence of dosing regimen on risedronate efficacy measured by lumbar spine bone mineral density (BMD). Risedronate IR tablets in a daily dose of 5 mg were administered (1) under fasted conditions, at least 30 min before the first meal of the day, or (2) between meals, at least 2 h from a meal or 30 min before bedtime. Both studies provided similar observations. Both dosing regimens resulted in a significant increase from the baseline in lumbar spine BMD, however response to risedronate was smaller for flexible dosing. In the first study, an increase in lumbar spine BMD after 6 months was 2.9% for (1) regimen and 1.5% for (2) regimen, and in the second study after 12 months, 4.4 and 3.2% respectively. Kendler et al. [41] suggested that minor response to treatment in the flexible dosing group may be due to less than optimal risedronate absorption, when taken between meals, or poorer compliance to this regimen, especially in certain geographic regions. 

Agrawal et al. [42] also studied the efficacy of the flexible-dosing approach in a randomized, double-blind trial in 60 residents of the nursing home (NH). 31 of participants were weekly administered 30 mg of risedronate in IR tablets, between meals but at least 2 h from a meal, and 29 received placebo. The effect of the treatment was measured by the changes in the serum levels of bone turnover markers (e.g., bone-specific alkaline phosphatise—BSAP). Risedronate administered in-between-meal schedule significantly reduced serum BSAP after 6 weeks of study relative to the control group, but this effect was not maintained after 12 weeks. Agrawal et al. [42] provided possible reasons for the ineffectiveness of flexible-dosing regimen in NH residents, such as delayed gastric emptying in the elderly, leading to impaired absorption of risedronate, when taken between meals, or high prevalence of vitamin D insufficiency that may contribute to the increase of bone turnover. 

##### Delayed-Release (DR) Tablets

Enteric-coated DR risedronate formulation was developed with the addition of ethylenediaminetetraacetic acid (EDTA), the chelator of metal ions. As a result, di- and trivalent cations in food may be preferentially bound by EDTA instead of risedronate, which eliminates the need for fasting and improves risedronate bioavailability, when compared with IR formulation [14]. For example, 35 mg of risedronate in DR tablet taken after a high-fat breakfast was found to have similar bioavailability as the same dose in IR tablet administered 4 h before a meal, and 2–4-fold greater bioavailability than the IR tablet administered 30 min before breakfast [25].

In a recent randomized, double-blind, placebo-controlled study of 68 healthy post-menopausal women, Fukase et al. [43] investigated the influence of dosing regimens on pharmacokinetic and pharmacodynamic parameters of risedronate in DR tablets. A single dose of 37.5 mg was administered (1) under fasting conditions—a reference regimen, (2) 30 min before a meal, (3) immediately after a meal, or (4) 30 min after a meal. The meal consisted of 492 kcal and contained 4% of fat. When compared to fasting conditions, taking risedronate DR tablets immediately after a meal resulted in a significant decrease of AUC_0–∞_ and C_max_ (by 58 and 69%, respectively). However, pharmacokinetic and pharmacodynamic profiles for regimen (2), (3), and (4) were comparable, hence Fukase et al. suggested that DR formulation can be taken independently from meals. 

Similar results were obtained in a cross-over pharmacokinetic study, where administration of DR tablet with high-fat breakfast resulted in a 30% decrease of bioavailability, compared to fasting state [25]. However, it should be noted that when DR tablets are taken on an empty stomach, an 8–9% increase in gastrointestinal adverse events may occur [25]. A possible solution to this problem could comprise administering risedronate after dinner. In a separate study, taking DR tablets in that regimen improved bioavailability by 87%, compared to the administration with breakfast. Nevertheless, the data for the safety and efficacy of taking risedronate DR tablets at bedtime is scarce [25].

In a randomized 2-year study of postmenopausal women with osteoporosis, McClung et al. [44,45] compared the efficacy of weekly administered 35 mg risedronate in DR tablets in two dosing regimens: at least 30 min before breakfast (BB), or following breakfast (FB) with 5 mg IR tablets taken daily while fasting. The effectiveness of the treatment was measured by changes in lumbar spine BMD. After 1 year of the study [44], the mean percent changes in lumbar spine BMD for 767 women were 3.4% in the 35 mg DR BB group, 3.3% in the 35 mg DR FB group, and 3.1% in the 5 mg IR daily group. After 2 years of treatment [45], the mean percent changes in lumbar spine BMD for 722 women were higher in all groups: 5.4% in the DR BB weekly group, 5.5% in the DR FB weekly group, and 4.4% in the IR daily group. McClung et al. concluded that weekly treatment with risedronate 35 mg DR is at least as effective as daily administered risedronate 5 mg IR, and DR formulation can be taken either before or after breakfast with similar efficacy.

On the contrary, a recent randomized phase II/III double-blind study [46] in Japanese patients with involutional osteoporosis revealed that the effectiveness of risedronate DR therapy may significantly differ, depending upon dosing regimen and formulation. Two doses of DR risedronate—25 and 37.5 mg were administered monthly for 1 year: (1) under fasting conditions, (2) concomitantly with breakfast, or (3) 30 min after breakfast. The efficacy of treatment was measured by changes in lumbar spine BMD and compared with daily administered IR 2.5 mg risedronate. At the end of the study, for DR 25 mg, percentage changes in lumbar spine BMD for regimens (1), (2), (3) were as follows: 3.82, 3.36, 3.93%, for DR 37.5 mg: 4.81, 4.11, 4.36%, respectively, and for IR 2.5 mg: 5.07%. Results indicated that risedronate ingestion immediately with breakfast is the least effective, and the non-inferiority of treatment with IR and DR formulations cannot be declared. 

Although most of the concerned studies indicate that DR formulation can be administered while eating, without significant changes in bioavailability and efficacy, still the intake of supplements containing di- and trivalent cations should be delayed. In a cross-over study on 101 postmenopausal women, the bioavailability of a single 35 mg dose of risedronate DR tablets was evaluated, when taken following breakfast, with or without the supplement containing 600 mg of elemental calcium and 400 IU of vitamin D. Concomitant intake of risedronate and calcium-containing supplement reduced drug absorption by 38% [25]. 

#### 3.1.6. Ibandronate

Ibandronate (syn. ibandronic acid, ibandronate sodium) for oral use is available in tablets (*Bonviva*, *Bondronat*), taken at monthly intervals. The mean oral bioavailability is approximately 0.6% for a dose of 2.5 mg [26].

In a pharmacokinetic study, the bioavailability of ibandronate was found to be dramatically reduced (by 90%), when 150 mg tablet was ingested concomitantly with a standard breakfast, relative to the fasting state [26]. Ingesting ibandronate 2 h after a standard meal or 30 min before breakfast significantly reduced the bioavailability as well (by 75% and 30%, respectively) [47]. No meaningful reduction in ibandronate bioavailability was observed for administration at least 60 min before breakfast [26,47]. 

In single-centre clinical trials, Nakai et al. [48] investigated the influence of fasting intervals on ibandronate bioavailability measured as AUC_0–∞_. In the first study, 24 healthy postmenopausal women were administered 2.5 mg ibandronate tablets 30 or 60 min before breakfast. Measured AUC_0–∞_ were 1.12 ± 0.95 and 1.40 ± 0.77 ng·h/mL, respectively. In the second study, 24 healthy men and postmenopausal women were given 50 mg ibandronate tablets. Measured AUC_0–∞_ values were 11.1 ± 23.5 ng·h/mL for 30-min fasting interval, and 16.0 ± 15.6 ng·h/mL for 60-min fasting interval. Nakai et al. concluded that shortening the fasting period from 60 to 30 min may result in lower ibandronate bioavailability, and hence recommended delaying food intake for 1 h after drug administration. 

In a 48-week, multi-centre, open-label study, Tanko et al. [49] examined whether different dosing regimens may influence the effectiveness of ibandronate treatment, measured by percentage changes in lumbar spine BMD from baseline. 184 postmenopausal women were randomly assigned to administer 2.5 mg ibandronate tablets 30 or 60 min before their standard breakfast. At the end of the study, significant increases in lumbar spine BMD were observed in both groups, however, in a group where a 60-min fasting interval was maintained, the mean change in the lumbar spine BMD was higher than in a group with a 30-min fasting interval (4.95 vs. 3.07%, respectively). Taking it into consideration, Tanko et al. suggested that fasting interval after ingesting ibandronate can be reduced to 30 min, however increasing drug dose may be required to maintain the efficacy of treatment. 

#### 3.1.7. Minodronate

Minodronate (syn. minodronic acid) is approved for the treatment of osteoporosis in Japan. Among available oral bisphosphonates, it is the strongest inhibitor of bone resorption, hence it can be administered once a month [50]. Bioavailability of minodronate after oral administration was calculated to be 1.21% [51]. 

In an open-label study of 12 healthy volunteers, Zhou et al. [40] investigated the influence of meals on minodronate absorption. A single dose of 4 mg was administered while fasting or 30 min before a high-fat breakfast (Table 1, Set No 6) consisted of 400 mg of elemental calcium. Taking minodronate 30 min before breakfast reduced AUC_0–∞_ and C_max_ by 72 and 55%, respectively, and increased t_max_ from 0.75 to 2.15 h relative to fasted conditions. These results indicate a necessity to administer minodronate while fasting. 

#### 3.1.8. Etidronate

Etidronate (syn. etidronic acid, etidronate disodium) is available in tablets (*Didronel*) taken in 90-days cycles: daily for 14 days, followed by calcium supplementation for the remaining 76 days. Although etidronate is no longer recommended in American College of Physicians guidelines for the treatment of osteoporosis [13], and it is not FDA-approved for this indication as well, in some countries etidronate use is still considered as the second-line therapy for osteoporosis [52].

The oral bioavailability of etidronate is approximately 3% [27]. A single-dose study on 10 healthy volunteers revealed that administering 400 mg etidronate during breakfast may reduce drug absorption to zero [53]. 

In a retrospective study of 110 patients with osteoporosis, Cook et al. [54] assessed the influence of etidronate dosing regimen on treatment effectiveness, measured by changes in BMD. Patients were administered etidronate (1) after waking up, (2) at bedtime, or (3) during the day, and maintained 2 h fasting intervals before and after drug ingestion. After approximately 2.6 years of study, no significant differences in percentage change in BMD were found between the three groups, indicating that the dosing regimen of etidronate does not affect the efficacy of therapy. 

Nevertheless, in another study of 70 patients treated with etidronate, Ryan et al. [55] revealed that drug ingestion during the day, even with 2 h fast before and after dosing, may still result in significantly impaired treatment response (lower percentage changes in BMD), when compared to administration in the early morning or late evening. 

### 3.2. Selective Estrogen Receptor Modulators (SERMs)

Selective estrogen receptor modulators (SERMs) act as agonists on estrogen receptors in bone—modulate skeletal homeostasis by diminishing the activity of osteoclasts and maintaining the function of osteoblasts [56]. SERMs decrease bone remodelling, prevent bone loss by increasing bone mineral density [56] and are recommended as the most beneficial in younger postmenopausal women with mild and moderate osteoporosis [57]. The most common side effects of treatment with SERMs are hot flashes, leg cramps, and an increased risk of deep vein thrombosis [56,57].

**Figure 3:**
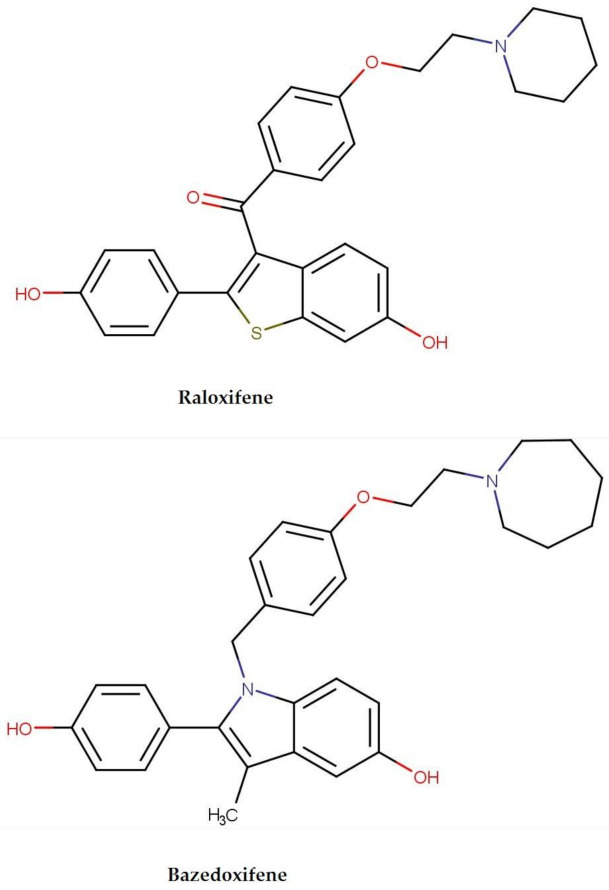
Chemical structures of SERMs discussed in the review.

The core structure of SERMs is based on 17β-estradiol template. Chemical structures of the discussed SERMs representatives are presented in Figure 3.

Both raloxifene and bazedoxifene belong to the II class of BCS—as lipophilic compounds they are hardly soluble in water and easily permeable by membrane cells. Low water solubility, together with the first-pass metabolism are the main factors negatively affecting oral bioavailability of these drugs [58].

#### 3.2.1. Raloxifene

Raloxifene is available in tablets (*Evista*), taken once daily. On average 60% of an oral dose is rapidly absorbed from the gastrointestinal tract, however, due to the extensive first-pass hepatic metabolism, absolute oral bioavailability is only 2% [59]. The presence of food does not influence raloxifene pharmacokinetics. Concomitant ingestion with a high-fat meal increased AUC and C_max_ by 16 and 28%, respectively, though these changes were considered insignificant [60]. 

#### 3.2.2. Bazedoxifene

Bazedoxifene is available in tablets taken daily, both alone (*Conbriza*) and in combination with conjugated estrogens (*Duavee*). The absolute oral bioavailability of bazedoxifene is on average 6% [61].

To examine the impact of food on bazedoxifene pharmacokinetic parameters, McKeand et al. [62] administered a single 10 mg bazedoxifene tablet to 84 healthy postmenopausal women, either while fasting, or 10 min before a high-fat breakfast. Concomitant intake with a meal reduced bazedoxifene C_max_ by 35%, but this change was considered insignificant; AUC for both dosing regimens were comparable. 

Other studies revealed that the administration of bazedoxifene with food may even slightly enhance drug absorption. After taking a 20 mg tablet with a high-fat meal, AUC and C_max_ increased by 28 and 22%, respectively, and after ingestion with a medium-fat meal, by 35 and 42%, correspondingly. However, these changes were found clinically irrelevant, and it was concluded that bazedoxifene may be taken regardless of food [61].

The influence of food was also investigated for bazedoxifene combined with conjugated estrogens. In a crossover study, 23 postmenopausal women were administered a single 20 mg/0.625 mg tablet either under fasting conditions, or with a high-fat, high-calorie meal. Ingestion with a meal increased AUC_0–∞_ by 25%, while C_max_ remained unchanged, suggesting that combined preparation can be administered without regard to meals as well [63].

### 3.3. Recomendations

Table 2 below presents the summary of the most important data and recommendations for appropriate intake of drugs employed in treating osteoporosis with regard to food. Knowledge on the subject, with special emphasis on oral bisphosphonates, is critical to educate patients professionally and effectively, which is the key intervention to enhance adherence to oral osteoporosis treatment.

## 4. Summary

A proper regimen of administration for medications used in oral osteoporosis therapy is crucial not only for the treatment effectiveness but also for minimizing the risk of adverse events and hence improving the patient’s quality of life. For the first-choice drugs, namely oral bisphosphonates, their combined administration with food, beverages, and dietary supplements containing multivalent cations, showed to have a deleterious effect on the bioavailability, and treatment efficacy. Changing drug formulation to delayed-release tablets may partially solve this problem, however more studies are needed. On the contrary, the effect of food on pharmacokinetic parameters of selective estrogen receptor modulators (SERMs) is clinically irrelevant. It is also worth noting that the data on the interactions of osteoporosis drugs with the individual food components is scarce, while the results of the existing studies suggests the importance of the problem. Thus, in our opinion, further in-depth studies are highly recommended, the results of which may complete the current strategy in enhancing the effectiveness of osteoporosis treatment. 

## Figures and Tables

**Table 1:** Detailed composition of the meals used in the studies described in this review.

Type of Meal	Food Items	Nutrition Facts	Energy	References
Alendronate				
Breakfast Set No 1	2 pieces of white toast, jelly/marmalade (20 g), 1 fried egg, 2 strips of bacon, and orange juice (250 mL)	70.6 g of carbohydrates, 6.3 g of fat, and 22.4 g of protein	Approx. 350 kcal	[22]
Breakfast Set No 2	1 piece of white toast, 2 pats of butter, 2 strips of bacon, 2 fried eggs, hash brown potatoes (2–4 oz), and whole milk (240 mL).	40.3 g of carbohydrates, 29 g of fat, and 89.4 g of protein	Approx. 665 kcal	[22]
Risedronate				
Lunch Set No 3	Smoked turkey, vegetable and beef soup with crackers, and whole wheat bread with lettuce (283 g), tossed salad (142 g) with light salad dressing (12 g), mayonnaise (15 mL), 2 canned peach halves, and skimmed milk (283 g)	104 g of carbohydrates, 19 g of fat, and 38 g of protein	Approx. 716 kcal	[39]
Breakfast Set No 4	2 slices of white toast, 2 pats of butter, 2 slices of bacon, hash brown potatoes (57 g), 2 eggs fried in butter, and whole milk (226 g)	50 g of carbohydrates, 46 g of fat, and 30 g of protein	Approx. 730 kcal	[39]
Dinner Set No 5	baked chicken breast (113 g), 1 baked potato, light gravy (28 g), 1 pat of margarine, 0.5 cup of apple sauce, 0.5 cup of carrot rounds, 1 peanut butter cookie, and lemonade (283 g)	103 g of carbohydrates, 16 g of fat, and 40 g of protein	Approx. 700 kcal	[39]
Minodronate				
Breakfast Set No 6	chicken drumstick, fried egg, hamburger, and milk	30% of carbohydrates, 55% of fat, 15% of protein	Approx. 900 kcal	[40]

**Table 2:** The summary of recommendations for appropriate intake of drugs employed in treating osteoporosis with regard to food.

Drug	Available Oral Formulations	Dosing Frequency	Recommendations with Regard to Food	Other Recommendations	References
Alendronate	tablets effervescent tablets oral solution jelly	daily or weekly	should be taken at least 30 min before breakfast should be taken at least 30 min before vitamins, mineral supplements, or antacids high in calcium, aluminum, or magnesium should be taken at least 2 h before iron preparations swallowing with coffee and juice should be avoided	tablets should be swallowed with a full glass of tap water effervescent tablets should be dissolved in a half a glass of tap water the oral solution should be taken with at least a quarter a glass of tap water jelly can be administered without water should be taken while standing or sitting in an upright position lying down for at least 30 min after administration should be avoided	[2,22,23,35,37,38]
Risedronate	immediate-release (IR) tablets	daily, weekly or monthly	should be taken at least 30 min before breakfast the flexible-dosing approach might be considered calcium, magnesium, and aluminum preparations should be administered at a different time of the day should be taken at least 2 h before iron preparations	should be swallowed with a full glass of tap water should be taken while standing or sitting in an upright position lying down for at least 30 min after administration should be avoided	[12,24,39,41,42]
	delayed-release (DR) tablets	Weekly	should be taken immediately following breakfast administration at bedtime might be considered calcium, magnesium, and aluminum preparations should be administered at a different time of the day should be taken at least 2 h before iron preparations	should be swallowed with at least half a glass of tap water should be taken while standing or sitting in an upright position lying down for at least 30 min after administration should be avoided	[14,25,43,44,45,46]
Ibandronate	tablets	Monthly	should be taken at least 1 h before breakfast should be taken at least 1 h before vitamins, mineral supplements, or antacids high in calcium, aluminum, or magnesium should be taken at least 2 h before iron preparations	should be swallowed with a full glass of tap water should be taken while standing or sitting in an upright position lying down for at least 60 min after administration should be avoided	[26,47,48,49]
Minodronate	tablets	Monthly	should be taken at least 30 min before breakfast	should be swallowed with a tap water should be taken while standing or sitting in an upright position lying down for at least 30 min after administration should be avoided	[40]
Etidronate	tablets	daily for 14 days, in 90-days cycles	should be taken at least 2 h before food (especially high-calcium products), early in the morning, or at bedtime should be taken at least 2 h before vitamins, mineral supplements, or antacids high in calcium, magnesium, aluminum, iron	should be swallowed with a full glass of tap water should be taken while standing or sitting in an upright position lying down immediately after administration should be avoided	[53,54,55]
Raloxifene	tablets	Daily	can be taken irrespectively of food		[60]
Bazedoxifene	tablets	Daily	can be taken irrespectively of food		[61,62,63]

## Data Availability

Data availability on the request.
